# Barium Promotes Anchorage-Independent Growth and Invasion of Human HaCaT Keratinocytes via Activation of c-SRC Kinase

**DOI:** 10.1371/journal.pone.0025636

**Published:** 2011-10-12

**Authors:** Nguyen Dinh Thang, Ichiro Yajima, Mayuko Y. Kumasaka, Shoko Ohnuma, Takeshi Yanagishita, Rumiko Hayashi, Hossain U. Shekhar, Daisuke Watanabe, Masashi Kato

**Affiliations:** 1 Units of Environmental Health Sciences, Department of Biomedical Sciences, College of Life and Health Sciences, Chubu University, Kasugai-shi, Aichi, Japan; 2 Department of Dermatology, School of Medicine, Aichi Medical University, Nagakute-cho, Aichi, Japan; 3 Voluntary Body for International Health Care in Chubu University, Kasugai-shi, Aichi, Japan; 4 Aichi Prefectural Institute of Public Health, Nagoya-shi, Aichi, Japan; The University of Kansas Medical Center, United States of America

## Abstract

Explosive increases in skin cancers have been reported in more than 36 million patients with arsenicosis caused by drinking arsenic-polluted well water. This study and previous studies showed high levels of barium as well as arsenic in the well water. However, there have been no reports showing a correlation between barium and cancer. In this study, we examined whether barium (BaCl_2_) may independently have cancer-related effects on human precancerous keratinocytes (HaCaT). Barium (5–50 µM) biologically promoted anchorage-independent growth and invasion of HaCaT cells *in vitro*. Barium (5 µM) biochemically enhanced activities of c-SRC, FAK, ERK and MT1-MMP molecules, which regulate anchorage-independent growth and/or invasion. A SRC kinase specific inhibitor, protein phosphatase 2 (PP2), blocked barium-mediated promotion of anchorage-independent growth and invasion with decreased c-SRC kinase activity. Barium (2.5–5 µM) also promoted anchorage-independent growth and invasion of fibroblasts (NIH3T3) and immortalized nontumorigenic melanocytes (melan-a), but not transformed cutaneous squamous cell carcinoma (HSC5 and A431) and malignant melanoma (Mel-ret) cells, with activation of c-SRC kinase. Taken together, our biological and biochemical findings newly suggest that the levels of barium shown in drinking well water independently has the cancer-promoting effects on precancerous keratinocytes, fibroblast and melanocytes *in vitro*.

## Introduction

Contamination of well drinking water with inorganic substances is a serious public health problem throughout the world. More than 25 million patients with arsenicosis have been reported in Bangladesh [1]. In Vietnam, 10 million patients with arsenicosis in the Red River Delta and 1 million patients with arsenicosis in the Mekong River Delta have been reported [2]. Epidemiological studies have revealed explosive increases in skin cancer in patients with arsenicosis [3]. Since many reports have provided evidence that arsenic enhances cellular malignant characteristics [3]–[5], arsenic in well water is thought to be a main cause of tumorigenesis. However, this evidence does not rule out the possibility that other elements as well as arsenic in well water contribute to carcinogenesis in humans.

Anchorage-independent growth, a hallmark of transformed cells [6]–[9], is a proliferative ability in the absence of adhesion to extracellular matrix (ECM) proteins and correlates closely with tumorigenesis [8]. Previous studies have revealed that activated c-SRC tyrosine kinase via autophosphorylation of tyrosine 418 and sequentially phosphorylated ERK were involved in anchorage-independent growth [7]. On the other hand, combined effects of increased cell motility and regulated proteolytic degradation of the matrix are necessary in tumor invasion, a hallmark of malignancy grade [10], [11]. Membrane type 1 matrix metalloproteinase (MT1-MMP) plays crucial roles in tumorigenesis through invasive growth of a tumor [4]. MT1-MMP expression and activity are related to ERK and are regulated by activities of c-SRC and focal adhesion kinase (FAK) [10], [12]. Thus, c-SRC, FAK, ERK and MT1-MMP play crucial roles in activities of anchorage-independent growth and/or invasion.

Barium is an ordinary inorganic substance in well drinking water and is classified as a low-toxic element at present [13]. To our knowledge, there are no reports showing barium is correlated with cancer *in vitro* and *in vivo*. In this study, we found that 2.5–5 µM of barium, which are common concentrations in drinking well water samples in various countries [14]–[16], may independently have cancer-promoting effects on precancerous keratinocytes, fibroblast and melanocytes *in vitro*.

## Results

### High concentration of barium in arsenic-polluted well water in both Bangladesh and Vietnam

**Figure 1:**
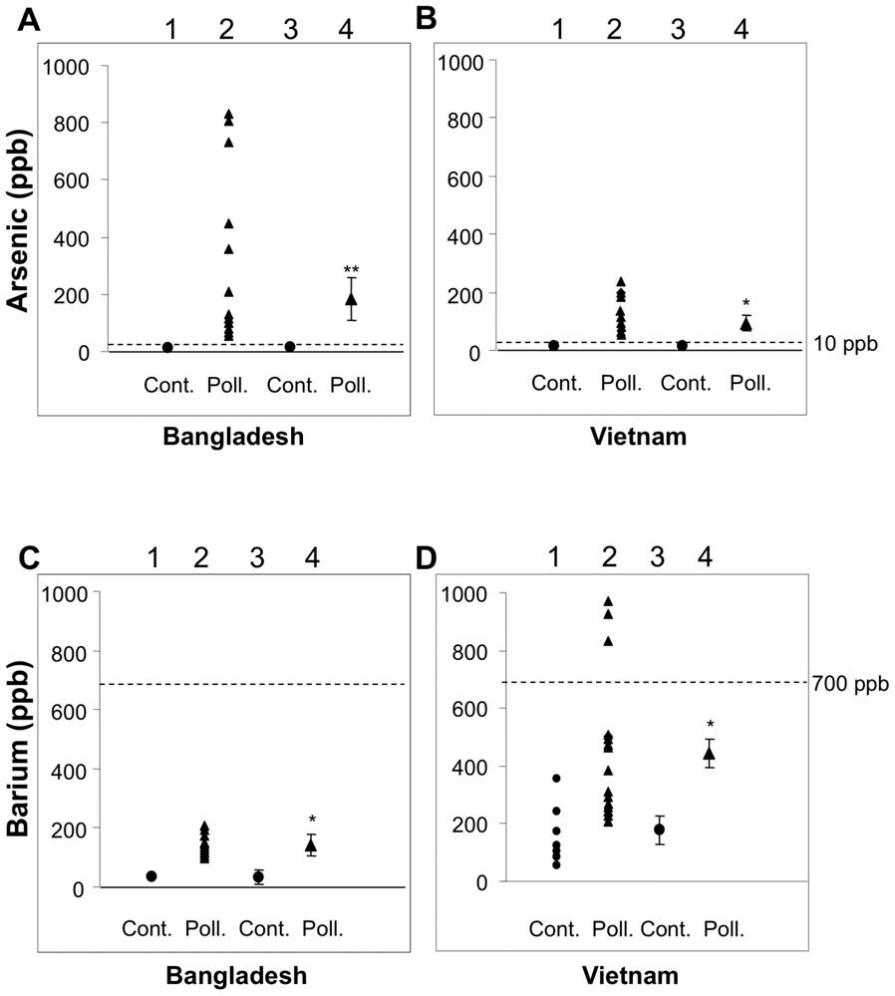
Levels of arsenic and barium in well water samples from Bangladesh and Vietnam. A–D, Arsenic (A, B) and barium (C, D) concentrations in well water samples from Bangladesh (A, C) and Vietnam (B, D) are presented. Value in each sample (lanes 1 and 2 in A–D) and mean ± SE (lanes 3 and 4 in A–D) are presented for samples from arsenic-polluted areas at Jessore in Bangladesh (lanes 2 and 4 in A and C) and Mekong Delta in Vietnam (lanes 2 and 4 in B and D) and control areas at Dhaka in Bangladesh (lanes 1 and 3 in A and C) and Ho Chi Minh in Vietnam (lanes 1 and 3 in B and D). * and **, Significantly different (*, p<0.05; **, p<0.01), respectively) from the control by the Mann-Whitney *U* test.

We first examined the concentrations of arsenic in well water at Samta, Jessore in Bangladesh, where patients with cancer are explosively increasing [17], [18]. The mean concentration of barium in arsenic-polluted well water was 5-fold higher than that in control well water without arsenic pollution at Dhaka in Bangladesh (Figure 1A and 1C). The mean concentration of barium in arsenic-polluted well water from Mekong Delta was 2.5-fold higher than that in well water from the control area without arsenic pollution at Ho Chi Minh in Vietnam (Figure 1B and 1D). Barium concentration beyond the level of WHO health-based guidelines (700 ppb) was observed in well water from Mekong Delta in Vietnam (Figure 1D). Previous reports also showed high levels of barium in well water in arsenic-polluted areas [14], [15]. These results suggest high concentrations of barium in arsenic-polluted well water in Bangladesh and Vietnam.

### Barium-mediated promotion of cellular anchorage-independent growth and invasion in HaCaT keratinocytes

**Figure 2:**
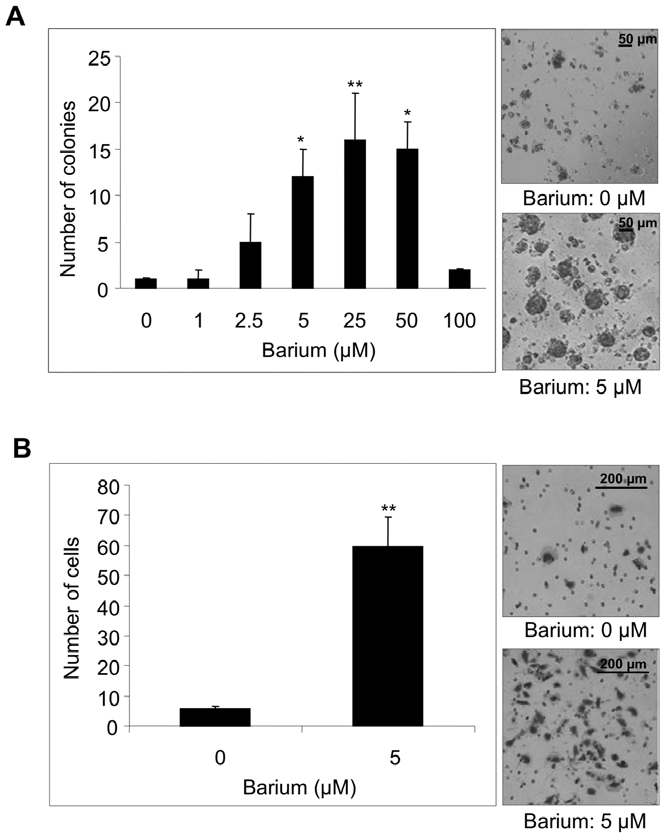
Effects of barium on anchorage-independent growth and invasion of HaCaT cells. A, Anchorage-independent growth of HaCaT cells treated with 0–100 µM of barium was evaluated by the colony formation assay. Level of anchorage-independent growth is presented as number of colonies in a graph (left) and photographs (right). B, Number of invading HaCaT cells treated with 0 or 5 µM of barium in the invasion assay is presented in a graph (left) and photographs (right). * and **, Significantly different (*, p<0.05; **, p<0.01) from the control by the Kruskal-Wallis test (A) and Mann-Whitney *U* test (B).

We next examined whether barium has cancer toxicity in human nontumorigenic keratinocytes (HaCaT cells) [19]. There was no morphological change in cells treated with 5–50 µM (686–6,860 ppb) barium. However, 5–50 µM of barium significantly promoted anchorage-independent growth of HaCaT keratinocytes (Figure 2A). The same concentration of barium also promoted anchorage-dependent growth, proliferative ability with adhesion to ECM proteins, in HaCaT cells (Figure S1). Furthermore, barium (5 µM) significantly promoted invasion of HaCaT cells (Figure 2B).

### Barium-mediated activation of c-SRC, FAK, ERK and MT1-MMP in HaCaT keratinocytes

**Figure 3:**
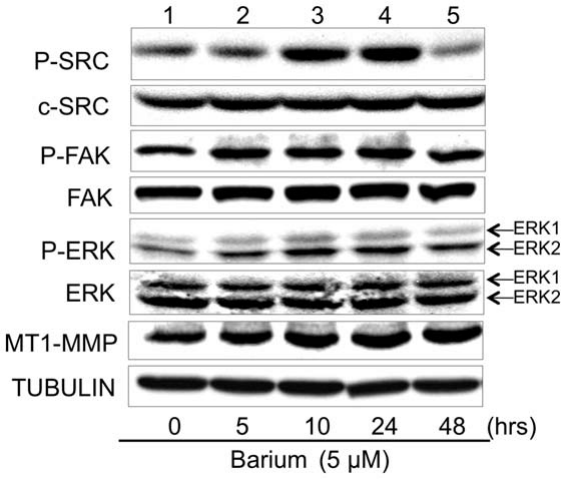
Levels of phosphorylation and protein expression of growth- and invasion-regulatory molecules in HaCaT cells treated with barium. Phosphorylated levels of c-SRC (P-SRC), FAK (P-FAK) and ERK (P-ERK) and protein expression levels of c-SRC, FAK, ERK and MT1-MMP in HaCaT cells treated with 5 µM barium for 0–48 hours (lanes 1–5) are presented. TUBULIN protein expression levels are presented as an internal control.

We next examined the molecular mechanism of barium-mediated cellular proliferation and invasion. In accordance with previous reports [20], [21], activities and expressions of SRC-related signaling molecules in HaCaT keratinocytes before treatment with barium (lane 1 in Figure 3) were detectable. This may be because HaCaT cells are not primary cultured normal keratinocytes and have precancerous characteristics [22]. Treatment of HaCaT keratinocytes with 5 µM barium for 10 hours more strongly enhanced the phosphorylated level of c-SRC than that of ERK (Figure 3). ERK phosphorylation level may be modulated by signal transduction molecules that are between c-SRC (upstream) and ERK (downstream) [23]. Phosphorylation level of c-SRC, but not that of ERK, was decreased at 48 hours after barium stimulation (lane 5 in Figure 3). Since continuous activation of c-SRC is linked to malignant transformation [23]–[25], the intracellular mechanism for downregulation of c-SRC activity might work earlier than that of ERK potentially sited downstream of c-SRC. Barium also increased phosphorylated levels of FAK (Figure 3) in HaCaT keratinocytes. Although our anti-MT1-MMP antibody could detect both proenzyme and active forms, no active form of MT1-MMP was detectable in HaCaT keratinocytes in the presence or absence of 5 µM barium (Figure 3). However, levels of the proenzyme of MT1-MMP were increased after barium stimulation (Figure 3). Our results partially correspond to results of previous studies showing no detection of the active form of MT1-MMP in constitutive HaCaT cells [26], [27].

### Inhibition of barium-mediated promotion of anchorage-independent growth and invasion in HaCaT keratinocytes by a SRC inhibitor

**Figure 4:**
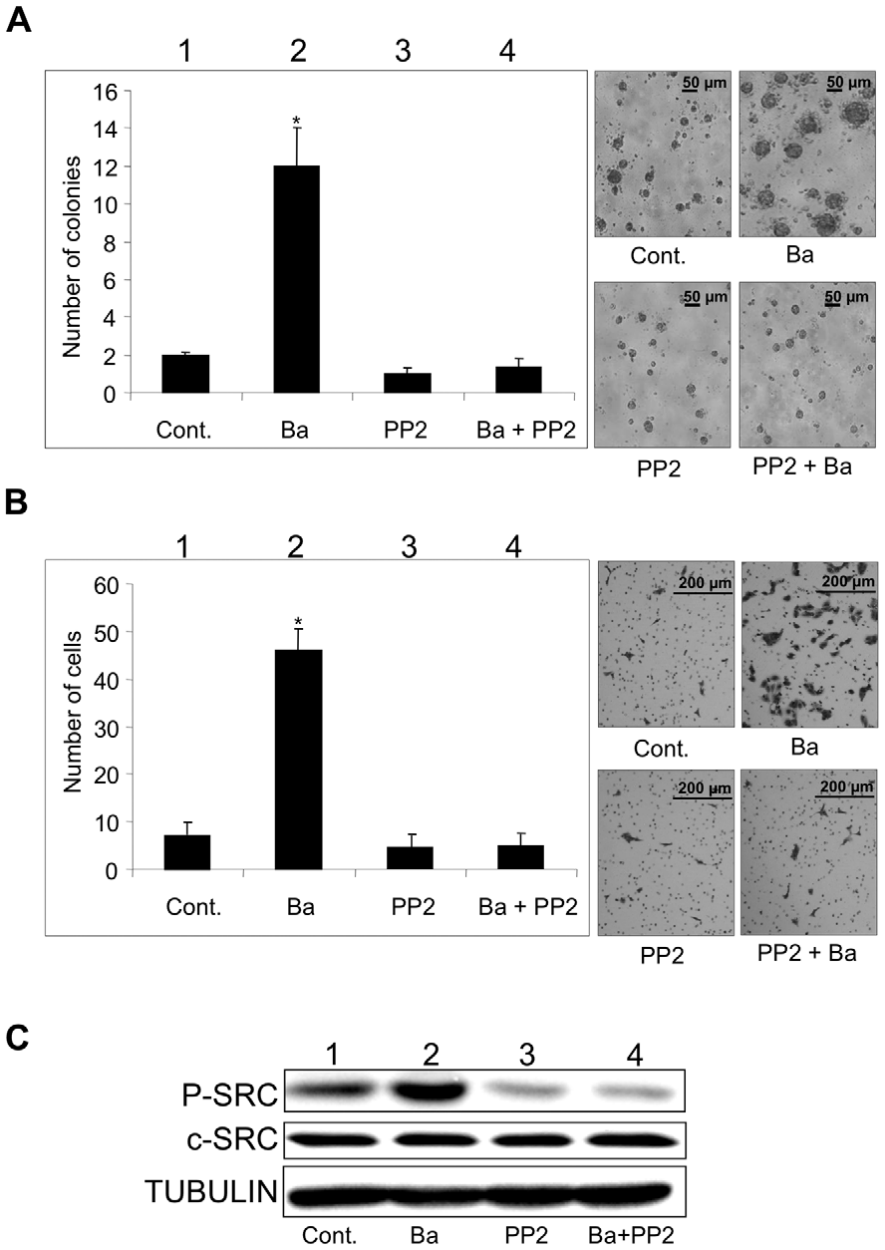
Effects of c-SRC kinase activity on barium-mediated anchorage-independent growth and invasion of HaCaT cells. A and B, Number of colonies by the colony formation assay (A) and number of invading cells by the invasion assay (B) are presented in a graph (left) and photographs (right). C, Phosphorylated levels of c-SRC (P-SRC) and protein expression levels of c-SRC (C) in HaCaT cells are presented. Cells treated with 5 µM of barium (lane 2), 1 µM of the SRC inhibitor PP2 (lane 3), 5 µM of barium and 1 µM of PP2 (lane 4) and nil control (lane 1) for 24 hours are presented. * Significantly different (*, p<0.05) from the control by the Kruskal-Wallis test.

Since c-SRC has been reported to be potentially sited upstream of FAK, ERK and MT1-MMP [10], [12] and might be associated with barium-mediated anchorage-independent growth and invasion (Figure 3), we next examined the effect of an SRC inhibitor of protein phosphatase 2 (PP2) on barium-mediated anchorage-independent growth and invasion of HaCaT cells (Figure 4). Barium (5 µM) again increased anchorage-independent growth and invasion with increase in phosphorylated levels of c-SRC kinase (lanes 1 and 2 in Figure 4A–C). PP2 alone (1 µM) slightly suppressed constitutive anchorage-independent growth and invasion of HaCaT keratinocytes with downregulation of c-SRC kinase activity (lanes 1 and 3 in Figure 4A–C). However, the difference was not statistically significant because basal levels of the constitutive growth and invasion of nontumorigenic HaCaT keratinocytes were limited. Barium-mediated anchorage-independent growth and invasion were blocked by treatment with PP2 with decrease in barium-mediated c-SRC kinase activation (lanes 1–4 in Figure 4A–C). We further confirmed the effect of c-SRC on barium-mediated modulation of invasion using c-SRC small interfering RNA (siRNA) (supplementary Method S1) in HaCaT cells. Barium-mediated increase of invasion was again blocked in the c-SRC depleted HaCaT cells (Figure S2).

### Barium-mediated promotion of cellular anchorage-independent growth and invasion with activation of c-SRC kinase in nontumorigenic fibroblasts and melanocytes

**Figure 5:**
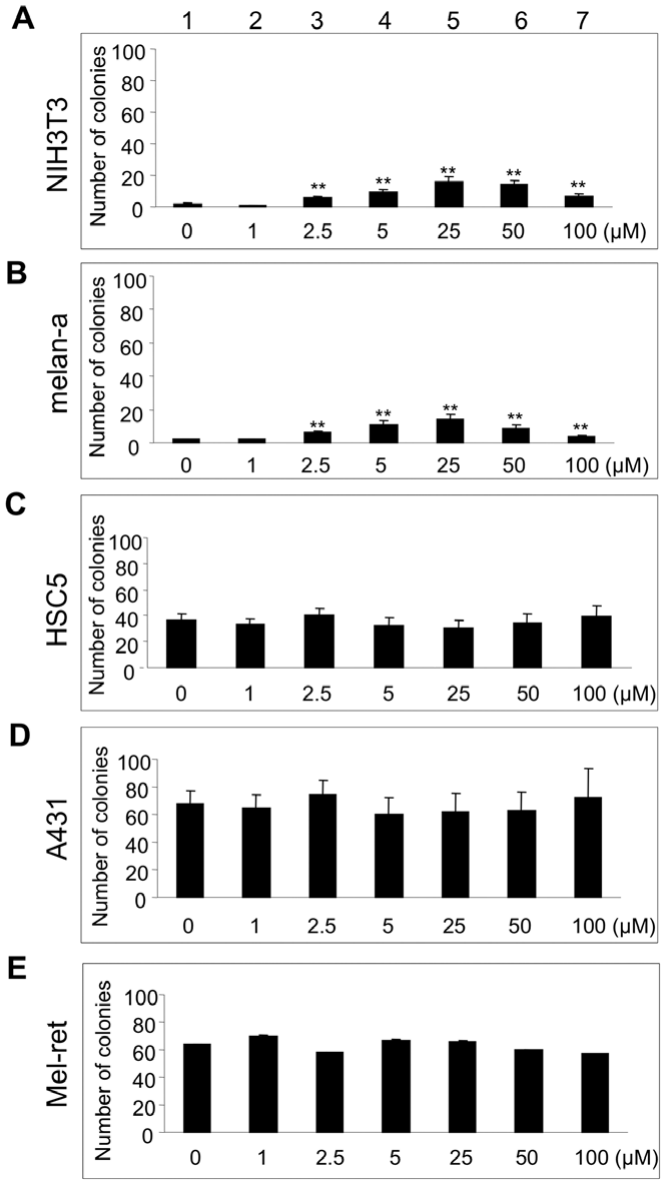
Effects of barium on anchorage-independent growth of nontumorigenic and transformed cells. Number of colonies in fibroblasts (NIH3T3; A), immortalized nontumorigenic melanocytes (melan-a; B), cutaneous squamous cell carcinoma cells [HSC5 (C) and A431 (D)] and malignant melanoma cells (Mel-ret; E) treated with 0–100 µM (lanes 1–7) of barium in the colony formation assay are presented. **, Significantly different (p<0.01) from the control by the Kruskal-Wallis test.

**Figure 6:**
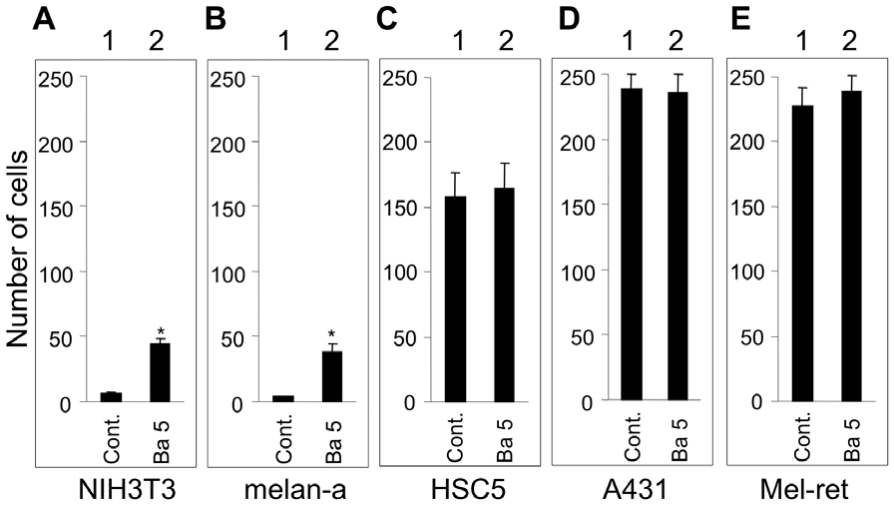
Effect of barium on invasion of nontumorigenic and transformed cells. Numbers of invading NIH3T3 (A), melan-a (B), HSC5 (C), A431 (D) and Mel-ret (E) cells treated with 0 µM (lane 1) or 5 µM (lane 2) of barium in the invasion assay are presented. *, Significantly different (*, p<0.05) from the control by the Mann-Whitney *U* test.

**Figure 7:**
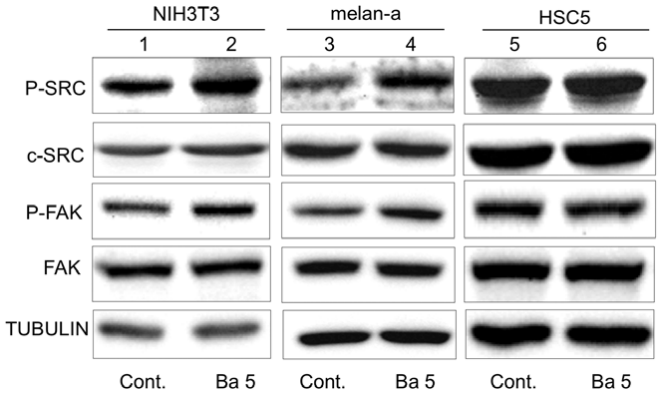
Levels of phosphorylation and protein expression of c-SRC and FAK in NIH3T3, melan-a and HSC-5 cells treated with barium. Phosphorylation (P-SRC and P-FAK) and/or protein expression (c-SRC and FAK) levels of c-SRC, FAK and TUBULIN in NIH3T3 (lanes 1 and 2), melan-a (lanes 3 and 4) and HSC-5 (lanes 5 and 6) cells treated with 0 µM (lanes 1, 3 and 5) and 5 µM (lanes 2, 4 and 6) barium for 24 hours are presented. Protein expression level of TUBULIN is presented as an internal control.

We next examined whether barium also promotes cellular anchorage-independent growth and invasion in cells other than HaCaT keratinocytes. As shown in Figure 5A and 5B, barium (2.5–100 µM, lanes 3–7) significantly (p<0.01) promoted anchorage-independent growth of nontumorigenic NIH3T3 and melan-a cells. On the other hand, barium (1–100 µM, lanes 2–7) had no effect on cellular anchorage-independent growth of transformed HSC5, A431 and Mel-ret cells (Figure 5C–E). Again, 5 µM of barium significantly (p<0.05) promoted invasion of NIH3T3 and melan-a cells but not HSC5, A431 and Mel-ret cells (Figure 6A–E). Correspondingly, c-SRC and FAK activities in nontumorigenic NIH3T3 (lanes 1 and 2 in Figure 7) and melan-a cells (lanes 3 and 4 in Figure 7), but not those in transformed HSC5 (lanes 5 and 6 in Figure 7), A431 (data not shown) and Mel-ret cells (data not shown), were promoted by 5 µM of barium.

## Discussion

To our knowledge, there are no reports showing barium as a carcinogen *in vitro* and *in vivo* [13], [28]–[30]. Previous studies *in vivo* showed increased blood pressure by vasoconstriction in mice treated with 100,000 ppb of barium for 16 months [29]. A significant decrease in survival rate with lymphoid depletions in the spleen, thymus and lymph nodes has been reported in mice treated with 2,500,000 ppb of barium for 15 months [13]. However, 2,000,000 ppb of barium had no effects on body weight, renal function and reproduction in rats and mice [30]. Thus, barium is thought to have low toxicity.

In this study, we first demonstrated concentrations of barium in well water in Bangladesh and Vietnam. We next demonstrated that 5 µM (686 ppb) of barium promotes anchorage-independent growth of nontumorigenic HaCaT keratinocytes. We also found that 2.5–100 µM (343–13,720 ppb) of barium promoted anchorage-independent growth of nontumorigenic NIH3T3 and melan-a cells. Since anchorage-independent growth is a hallmark of transformed cells [6]–[9], these results *in vitro* suggest that barium promotes tumorigenic transformation in various nontumorigenic cells. On the other hand, barium (1–100 µM) did not affect anchorage-independent growth of transformed HSC5, A431 and Mel-ret cells, suggesting limited effects of barium to promote anchorage-independent growth in transformed cells. Barium (5 µM) also promoted invasion of HaCaT, NIH3T3 and melan-a cells but not HSC5, A431 and Mel-ret cells *in vitro*. Since a high level of activity of invasion is a hallmark of high-grade malignancy [10], [11], our results suggest that barium specifically promotes malignant potential in nontumorigenic keratinocytes, fibroblast and melanocytes.

We further analyzed the mechanism of barium-mediated anchorage-independent growth and invasion. Barium (5 µM) activates c-Src, FAK, ERK and MT1-MMP in HaCaT keratinocytes. Barium also activates c-Src in NIH3T3 and melan-a cells. Since c-SRC and ERK kinase activities regulate anchorage-independent growth [7], these results suggest that barium-mediated promotion of anchorage-independent growth is associated with c-SRC and ERK pathway. In addition, c-SRC, FAK, ERK and MT1-MMP have been reported to play crucial roles in invasion [4], [10], [12]. Therefore, these results also suggest that barium-mediated upregulation of invasion is correlated with activation of c-SRC, FAK, ERK and MT1-MMP. Furthermore, our results showed that both a c-SRC kinase inhibitor and siRNA blocked barium-mediated anchorage-independent growth and invasion. Since FAK, ERK and MT1-MMP molecules have been reported to be potentially downstream of c-SRC kinase [31], [32], our results suggest that c-SRC kinase plays a key role in the barium-mediated promotion of malignant characteristics in nontumorigenic cells. Thus, not only the evidence of cell functions such as anchorage-independent growth and invasion but also the molecular basis analysis in this study suggests that barium has tumor-promoting effects. On the other hand, barium-mediated increase of FAK phosphorylation started earlier than that of c-SRC phosphorylation, although FAK was found as a substrate of c-SRC and was thought to be downstream of c-SRC [33]. Together with our results showing that enhancement of the protein level of FAK, but not that of c-SRC, by barium started 5 hours after stimulation, these results suggest that another mechanism in addition to the c-SRC/FAK pathway might work in the barium-mediated tumor-promoting effects.

Barium concentrations in nail and hair samples from Bangladeshi (n = 13) who drank arsenic-polluted well water as shown in Figure 1C were 2,242 ppb and 6,131 ppb, respectively (Kato et al. unpublished observation), and barium concentrations in nail and hair samples from Bangladeshi (n = 14) who drank control well water as shown in Figure 1C were 1,510 ppb and 2,831 ppb, respectively (Kato et al. unpublished observation). These results in human samples suggest that the barium concentrations of 2.5–5 µM (343–686 ppb) in this study are not extraordinary high. In fact, 2.5–5 µM of barium is less than the concentration in the WHO health-based guidelines (700 ppb = 5.1 µM).

Previous epidemiological studies revealed that skin cancer is explosively increasing in areas with arsenic-polluted well water [17], [18]. Previous environmental studies [14], [15] and results of this study showed high levels of barium in arsenic-polluted well water. These results indirectly suggest that barium in arsenic-polluted well water is associated with development of skin cancer. However, there is no direct epidemiological evidence of a higher incidence of skin cancer in people exposed to barium-polluted water. Therefore, skin cancer may develop due to the effect of arsenic rather than barium contamination in drinking well water.

In summary, we showed that treatment with 2.5–5 µM of barium enhances anchorage-independent growth and invasion of nontumorigenic keratinocytes, fibroblast and melanocytes *in vitro*. We then clarified that the barium-mediated promotion of anchorage-independent growth and invasion might be regulated through activation of c-SRC kinase. Thus, both biological and biochemical results may give us an opportunity to reconsider the risk of barium in drinking well water for skin cancer.

## Materials and Methods

### Barium concentration analysis

The levels of arsenic and barium in well water samples from Jessore (n = 15) and Dhaka (n = 45) in Bangladesh and Mekong Delta (n = 22) and Ho Chi Minh (n = 8) in Vietnam were measured by using an inductively coupled plasma-mass spectrophotometer (ICP-MS; 7500cx, Agilent Technologies Inc, CA) following the method previously described [34].

### Cell lines and culture conditions

Human nontumorigenic HaCaT keratinocytes [19], [22] (German Cancer Research Center ‘DKFZ’, Germany), human transformed keratinocytes HSC5 [35] (Health Science Research Resources Bank, Japan) and A431 [36] (Riken Bioresource Center, Japan) and murine malignant melanoma cells (Mel-ret) [37] (gifted by Dr. Masahide Takahashi of Nagoya University, Japan) were cultured in RPMI-1640 with 10% fetal bovine serum (FBS) on collagen-coated dishes. Murine nontumorigenic immortalized melanocytes (melan-a) [38] (gifted by Dr. Dorothy C Bennett of St George's Hospital Medical School, UK) and fibroblasts (NIH3T3) [7] (Riken Bioresource Center, Japan) were cultured in DMEM with 10% FBS on collagen-coated dishes. Up to 25 µM of barium (BaCl_2_; Wako) and 1 µM of protein phosphatase 2 (PP2: Calbiochem) had no effects on viability of these cells.

### Colony formation assay

Anchorage-independent growth was evaluated by the colony formation assay according to the method previously reported [39]. After cells had been preincubated with or without barium for 24 hours, 2.5×10^4^ cells were mixed with 2 ml of 0.36% soft agar in RPMI medium with or without barium, poured onto slightly solid 0.72% hard agar in RPMI medium, and then cultured for 3 weeks. Colonies exceeding 50 µm in diameter were counted and presented as an activity of anchorage-independent growth.

### *In vitro* invasion assay

After cells had been cultured in culture medium with 0.5% FBS for 12 hrs, they were treated with or without barium for 24 hours in culture media with 10% FBS. The cells were rinsed to remove barium and were subjected to an invasion assay in the absence of barium, according to the method described previously [10]. Briefly, 2×10^5^ cells in 300 µl culture medium with 0.5% FBS were applied to the matrigel-coated upper chamber of 8 mm in diameter (8 µm in pore size). Then the upper chambers were placed in 24-well culture plates containing 600 µl conditioned medium with 0.5% FBS to trigger invasion activity and were incubated for 12 hours. Invading cells were stained with hematoxylin and counted under a microscope.

### Immunoblot analysis

Immunoblot analysis was performed according to the method described previously [40]. Rabbit polyclonal first antibodies against phosphorylated tyrosine 418 in c-SRC (Sigma), phosphorylated threonine 202 in ERK1 and phosphorylated tyrosine 204 in ERK2 (Cell Signaling) and phosphorylated tyrosine 397 in FAK (Invitrogen) and anti-matrix metalloproteinase-14 (MT1-MMP) hinge region antibody (Millipore), mouse monoclonal first antibodies against alpha-tubulin (SIGMA), c-Src (Millipore), ERK1/2 (Cell Signaling) and FAK (Millipore) were used.

### Statistical analysis

Statistical analysis was performed following the method previously described [41]. We used the SPSS version 18 software package (SPSS Japan Inc.) for the above statistical analyses, and the significance level was set at p<0.05.

## Supporting Information

Figure S1 **Effect of barium on anchorage-dependent growth.** A and B, Morphology (A) and ratio of cell numbers (B) in HaCaT cells treated with 0–50 µM of barium (lanes 1–3) are presented. Cells stained with crystal violet were counted after culturing for 3 days in the presence or absence of barium. *, Significantly different (p<0.05) from the control by the Kruskal-Wallis test.
(TIF)

Figure S2 **Effects of c-Src siRNA on barium-mediated invasion of HaCaT cells.** A and B, number of invading cells by the invasion assay (A) are presented in graph (left) and photographs (right). Phosphorylated levels of c-SRC (P-SRC) and protein expression levels of c-SRC (B) in HaCaT cells are presented. Cells treated with 5 µM of barium (lane 2), 40 pmol/mL of the c-SRC siRNA (lane 3), 5 µM of barium and 40 pmol/mL of c-SRC siRNA (lane 4) and nil control (lane 1) for 24 hours are presented. **, Significantly different (p<0.01) from the control by the Kruskal-Wallis test.
(TIF)

Methods S1 *RNA interference* **:** Small interfering RNA (siRNA)-mediated depletion (knockdown) of c-SRC was performed with 21-nucleotide (5′-AAGCACUACAAGAUCCGCAAG-3′) synthetic duplexes (Hokkaido System Science Co. ltd). Cells were transfected with c-SRC siRNA or a 21-nucleotide control RNA (Invitrogen) using Lipofectamine RNAi MAX (Invitrogen) according to the manufacturer's protocol.
(DOC)
