# Supplementary figures and images for: Barium Promotes Anchorage-Independent Growth and Invasion of Human HaCaT Keratinocytes via Activation of c-SRC Kinase
Source: PLoS One. 2011 Oct 12;6(10):e25636. doi: 10.1371/journal.pone.0025636 (PMC3192110; doi:10.1371/journal.pone.0025636)

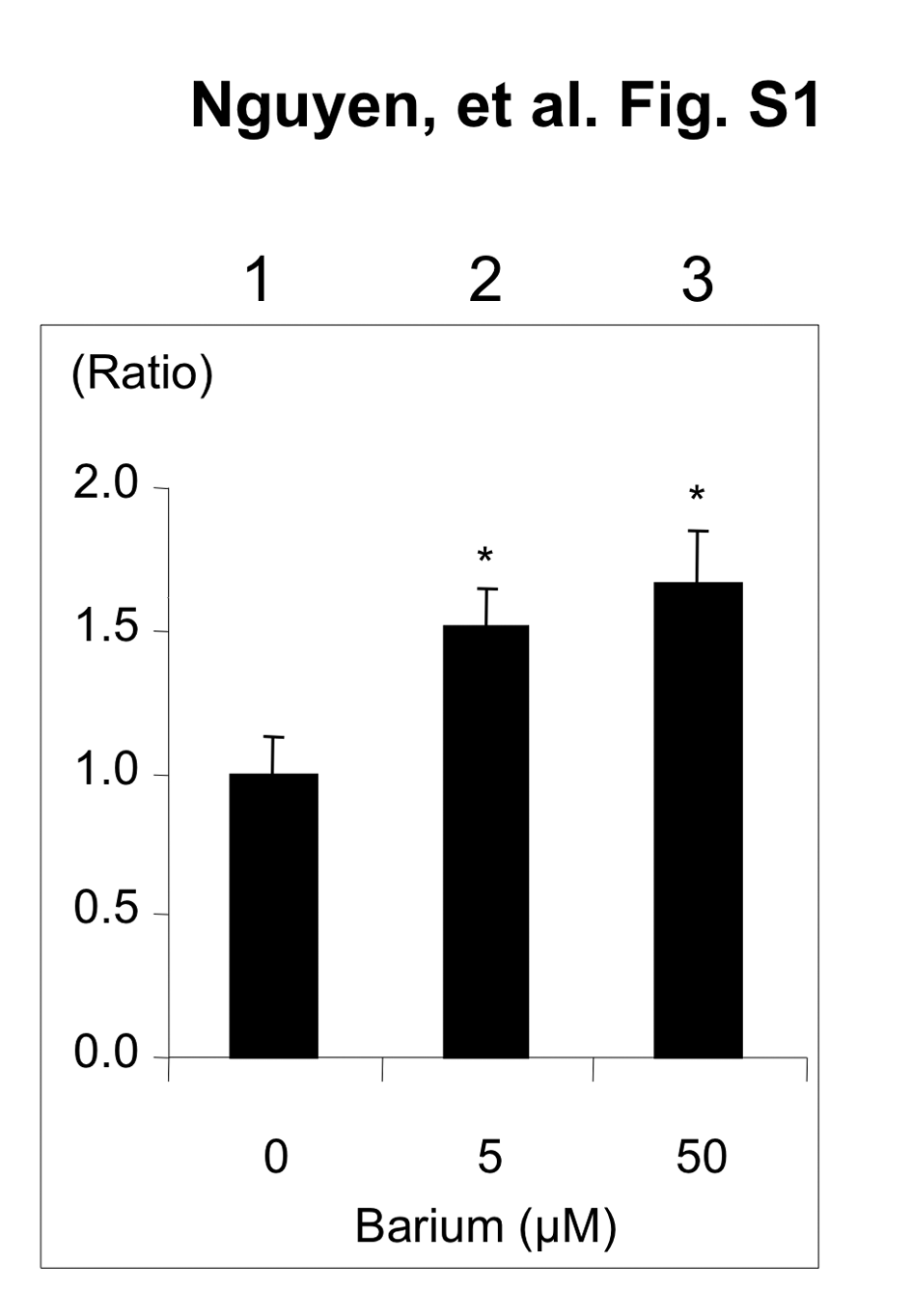

Supplement: Figure S1 — Effect of barium on anchorage-dependent growth. A and B, Morphology (A) and ratio of cell numbers (B) in HaCaT cells treated with 0–50 µM of barium (lanes 1–3) are presented. Cells stained with crystal violet were counted after culturing for 3 days in the presence or absence of barium. *, Significantly different (p<0.05) from the control by the Kruskal-Wallis test. (TIF) [file pone.0025636.s001.tif]

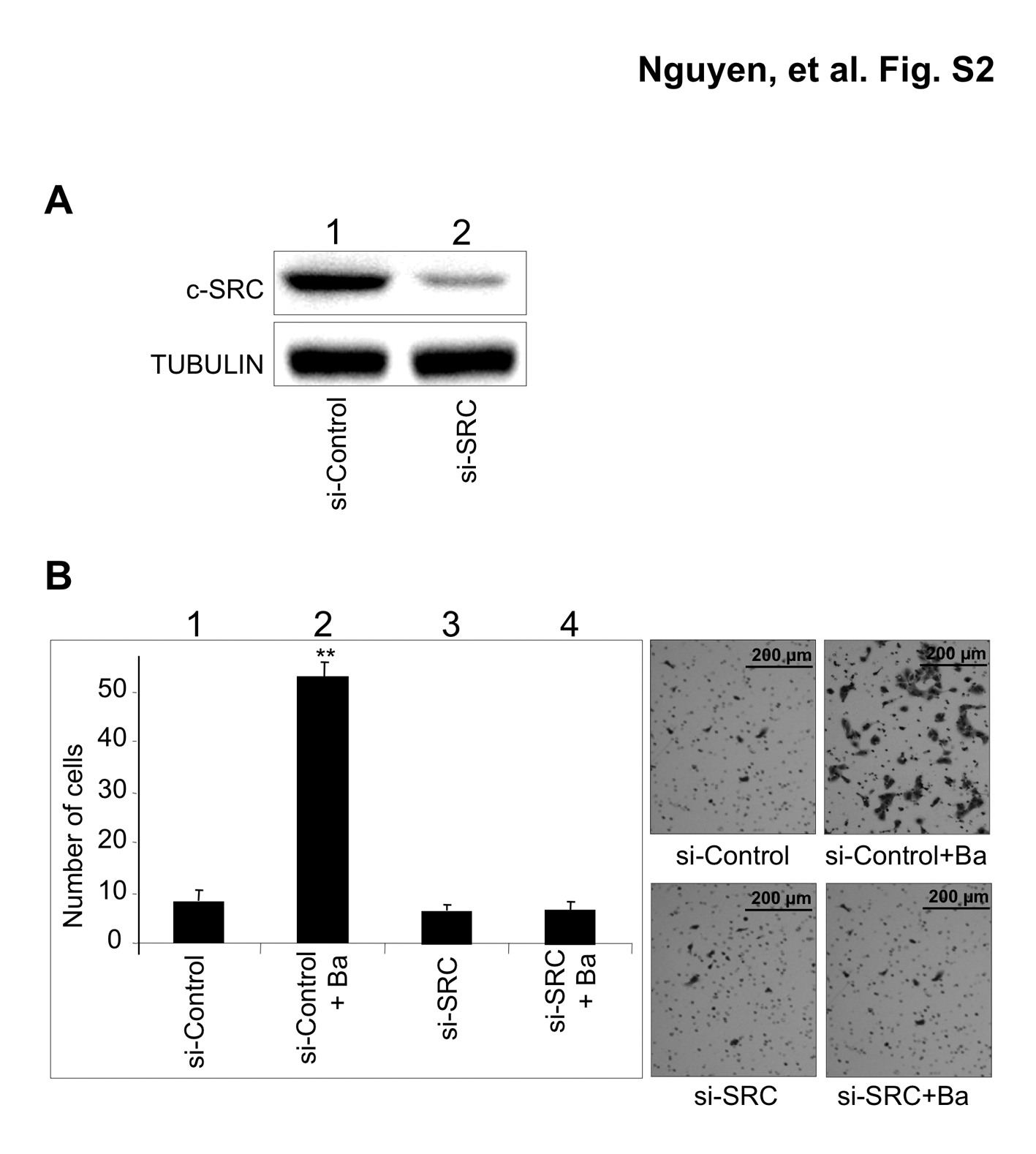

Supplement: Figure S2 — Effects of c-Src siRNA on barium-mediated invasion of HaCaT cells. A and B, number of invading cells by the invasion assay (A) are presented in graph (left) and photographs (right). Phosphorylated levels of c-SRC (P-SRC) and protein expression levels of c-SRC (B) in HaCaT cells are presented. Cells treated with 5 µM of barium (lane 2), 40 pmol/mL of the c-SRC siRNA (lane 3), 5 µM of barium and 40 pmol/mL of c-SRC siRNA (lane 4) and nil control (lane 1) for 24 hours are presented. **, Significantly different (p<0.01) from the control by the Kruskal-Wallis test. (TIF) [file pone.0025636.s002.tif]
