# Supplementary material for: Barium Promotes Anchorage-Independent Growth and Invasion of Human HaCaT Keratinocytes via Activation of c-SRC Kinase
Source: PLoS One. 2011 Oct 12;6(10):e25636. doi: 10.1371/journal.pone.0025636 (PMC3192110; doi:10.1371/journal.pone.0025636)
Supplement: Methods S1 — RNA interference : Small interfering RNA (siRNA)-mediated depletion (knockdown) of c-SRC was performed with 21-nucleotide (5′-AAGCACUACAAGAUCCGCAAG-3′) synthetic duplexes (Hokkaido System Science Co. ltd). Cells were transfected with c-SRC siRNA or a 21-nucleotide control RNA (Invitrogen) using Lipofectamine RNAi MAX (Invitrogen) according to the manufacturer's protocol. (DOC) [file pone.0025636.s003.doc]

**SUPPLEMENTARY METHOD**

**RNA interference**

Small interfering RNA (siRNA)-mediated depletion (knockdown) of c-SRC was performed with 21-nucleotide (5’-AAGCACUACAAGAUCCGCAAG-3’) synthetic duplexes (Hokkaido System Science Co. ltd). Cells were transfected with c-SRC siRNA or a 21-nucleotide control RNA (Invitrogen) using Lipofectamine RNAi MAX (Invitrogen) according to the manufacturer’s protocol.
